# NTPDase2+ Cells Generate Lingual Epithelia and Papillae

**DOI:** 10.3389/fgene.2012.00255

**Published:** 2012-11-27

**Authors:** Feng Li, Jie Cao, Mingliang Zhou

**Affiliations:** ^1^School of Medicine, Shanghai Jiao Tong UniversityShanghai, China; ^2^Monell Chemical Senses CenterPhiladelphia, PA, USA

**Keywords:** NTPDase2+ cell, lingual epithelia, lingual papillae, cell migration stream

## Abstract

The tongue epithelium is one of the most rapidly self-renewing tissues in adult mammals. Multiple stem cell populations are currently believed to exist in tongue epithelia. Keratin 14 (K14) positive cells differentiate into either lingual epithelia or lingual papillae, while ecto-nucleoside triphosphate diphosphohydrolase 2 (NTPDase2) is associated with neural stem cells and astrocyte-like cells ensheathing the migrating neuroblasts. Here, using a transgenic mouse expressing *rtTA* from the mouse *NTPDase2* promoter, we generated an inducible model by treatment with Doxycycline. By immunohistochemical analysis and *in situ* hybridization, we found exclusive expression of NTPDase2 in lingual epithelia and lingual papillae. Using inducible genetic cell fate mapping, we further showed that the NTPDase2+ cells generated lingual papillae and epithelia in the adult tongue. Finally, building on our previously proposed paradigm of cell migration stream, a model is further described here for lingual epithelia cell genesis. In short, the current results not only extend our understanding of the cell migration stream in lingual epithelia and lingual papillae, but they also support the concept of multiple stem cell populations in lingual epithelia and papillae.

## Introduction

**Figure 1:**
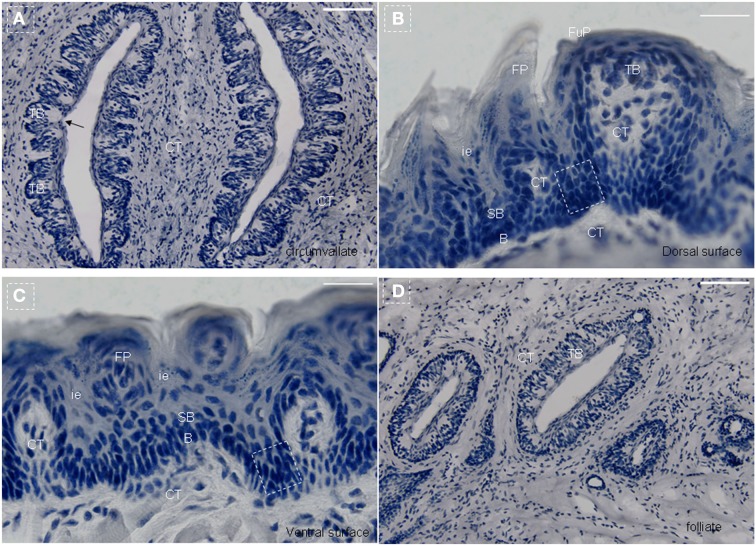
**Lingual papillae**. The mouse tongue contains numerous lingual papillae creating a specialized mucosa. The three main types are filiform, fungiform, and circumvallate (CV) papillae. A fourth type located at the edges of the tongue is the foliate papillae. Taste buds are present on fungiform, CV, and foliate papillae. **(A)** Horizontal section of CV papillae. **(B)** Horizontal section of foliate papillae. **(C)** Frontal section of dorsal surface in the tip of the tongue. **(D)** Frontal section of ventral surface in the tip of the tongue. Arrow, taste pore. Dotted frame indicates area between the arms of the U-shaped connective tissue core. B, basal cell layer; SB, suprabasal cell layer; CT, connective tissue; CV, circumvallate papillae; FP, filiform papillae; FuP, fungiform papillae; ie, interpapillary epithelia; TB, taste bud. Scale bar: 12 μm.

The mouse tongue contains numerous papillae creating a specialized mucosa. The three main types are circumvallate (CV; Figure 1A), fungiform (Figure 1B), and filiform papillae (Figures 1B,C). A fourth type located at the edges of the tongue is the foliate papillae (Figure 1D). Taste buds are present on fungiform (Figure 1B), CV (Figure 1A), and foliate papillae (Figure 1D; Ross and Pawlina, 2006). In all cases, they arise during development from the local surface epithelium rather than from neuronal or neural crest cells (Stone et al., 1995, 2002). Indeed, studies have shown that both the receptor cells of the taste buds and the stratified keratinized epithelium of the fungiform papillae in which they reside, as well as the numerous filiform papillae of the tongue, are all derived from the same simple epithelium present in the embryo (Farbman, 1965a; Baratz and Farbman, 1975). Similar to other epithelia, tongue epithelia need to constantly replace damaged or dead cells throughout the life of mammals. It has been shown that the turn-over time of taste receptors cells in adult mammals is approximately 10 days (Beidler and Smallman, 1965). For lingual epithelia in mice, this period is less than 3 days (Cameron, 1966). In general, the terminally differentiated cells of the epidermis cannot divide, and their replacement depends on less highly differentiated cells, i.e., stem cells.

Although lingual epithelia and papillae undergo continual turn-over, the existence of multipotent stem cells in the adult tongue has not yet been demonstrated. A previous study using pulse-chase tritiated thymidine suggested that long-term, self-renewing stem cells reside in the base of papillae. This model further showed that the transit amplifying (TA) and differentiating descendants move up along the basal lamina and exit into the superficial layers to be shed from the surface within approximately 5–7 days (Hume and Potten, 1976). In other study, a model was suggested for the location and niche of long-term stem cells in the tongue and further indicated multiple populations of bipotential progenitor cells located in the lingual epithelia (Okubo et al., 2006, 2009). Several studies have revealed that taste buds originate exclusively from the local epithelium with no contribution from neural crest, and the subsequent formation of the taste bud is reliant upon epithelial-mesenchymal interaction (Barlow et al., 1996; Barlow and Northcutt, 1997; Thirumangalathu and Barlow, 2009; Thirumangalathu et al., 2009). Recently, we proposed a model for the existence of a cell migration stream in the lingual epithelia and filiform papillae, consisting of multiple stem cell pools and differentiation cell pools. In particular, K14+ lineage cells contribute to the development of the lingual papillae and epithelia (Li and Zhou, 2012).

Ecto-nucleoside triphosphate diphosphohydrolase 2 (NTPDase2), which hydrolyzes extracellular nucleoside triphosphates to the respective diphosphates, is selectively associated with type-B cells, the stem cells of the subventricular zone (SVZ; Braun et al., 2003), and can also be associated with GFAP-, nestin-, and DCX-positive radial cells of the dentate gyrus (Shukla et al., 2005). In taste buds, NTPDase2 was found to co-localize with glial glutamate/aspartate transporter (GLAST), which is regarded as a marker of type I cells (Lawton et al., 2000; Bartel et al., 2006). Since then, NTPDase2+ cells have been considered as type I cells, having certain biological functions of supporting cells such as clearance of ATP due to the importance of ATP as an intercellular signaling molecule in taste buds (Finger et al., 2005; Bartel et al., 2006). In order to further characterize NTPDase2+ cells in this system, here we generated a Doxycycline inducible, NTPDase2-rtTA/TeTO-Cre/Rosa26R reporter system to carry out cell lineage mapping studies in the adult lingual epithelia and papillae. Our results not only extend our understanding of the cell migration stream in lingual epithelia and lingual papillae, but also support the concept of multiple stem cell populations in lingual epithelia and papillae.

## Results

**Figure 2:**
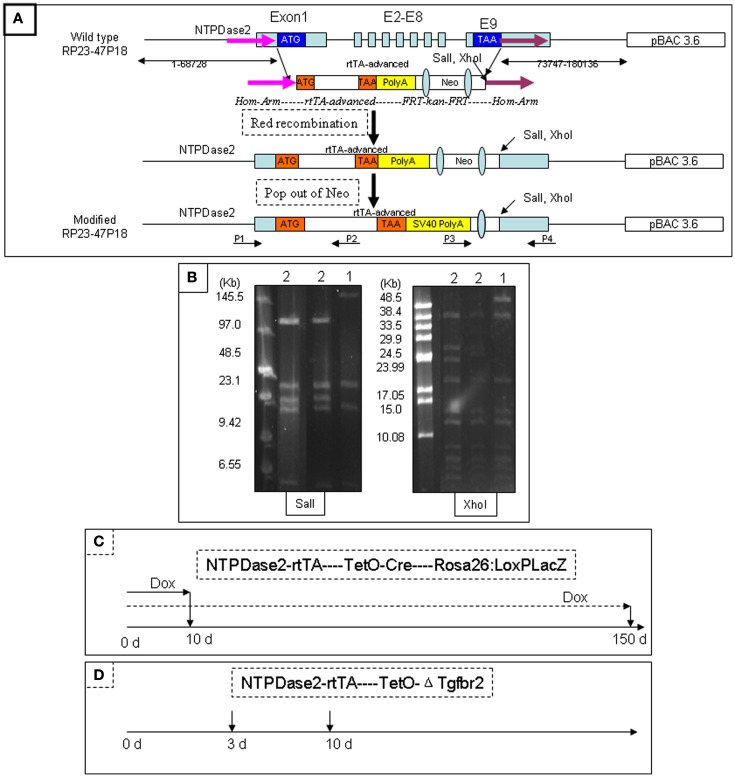
**Generation of NTPDase2-rtTA transgenic mice**. The BAC clone RP23-47P18 contains more than 70 kb of 3′ and 5′ sequences of the *NTPDase2* gene. The *rtTA* gene and its polyadenylation signal were inserted into the BAC clone RP23-47P18 by Red-mediated homologous recombination, replacing the coding sequence of the *NTPDase2* gene. *Sal*I and *Xho*I were introduced downstream of the *rtTA* gene. The transgenic mice were identified by tail-PCR using two pairs of oligonucleotides (P1/P2, P3/P4) specific for the *NTPDase2-rtTA* transgene. **(A)** Pulse-field gel electrophoresis (PFGE) was used to identify the intactness of the modified BAC clone. *Sal*I digestion released a 140 kb fragment containing the *NTPDase2* gene in the original BAC DNA. However, the 140 kb fragment was cut into two fragments, 33 and 107 kb, in the modified BAC DNA. *Xho*I digestion released a 56 kb fragment containing the *NTPDase2* gene in the original BAC DNA. The 56 kb fragment was cut into two pieces, 27 and 28 kb in the modified BAC DNA. (1) Original BAC DNA, RP23-47P18. (2) Modified BAC DNA. **(B)** The triple-transgenic mice (NTPDase2-rtTA-TetO-Cre-Rosa26: LoxPLacZ) were continually induced with Dox for 10 or 150 days. **(C)** The PTR transgenic mice contain the plasmid construct, which consists of the gene encoding enhanced green fluorescent protein (EGFP), a bidirectional tetracycline transactivator responsive promoter (*pBi*), and a truncated version of the *T*β*RII* gene with a c-myc epitope tag. In order to verify the function of rtTA, we crossed NTPDase2-rtTA transgenic mice with the PTR mice and obtained a double-transgenic strain. The binding of rtTA to the tetracycline responsive element (TetRE) and the induction of the transgene *EGFP*/Δ*Tgfbr2-c-myc* should only occur in the presence of Doxycycline. The double-transgenic mice were induced by adding Dox into the drinking water **(D)**.

**Figure 3:**
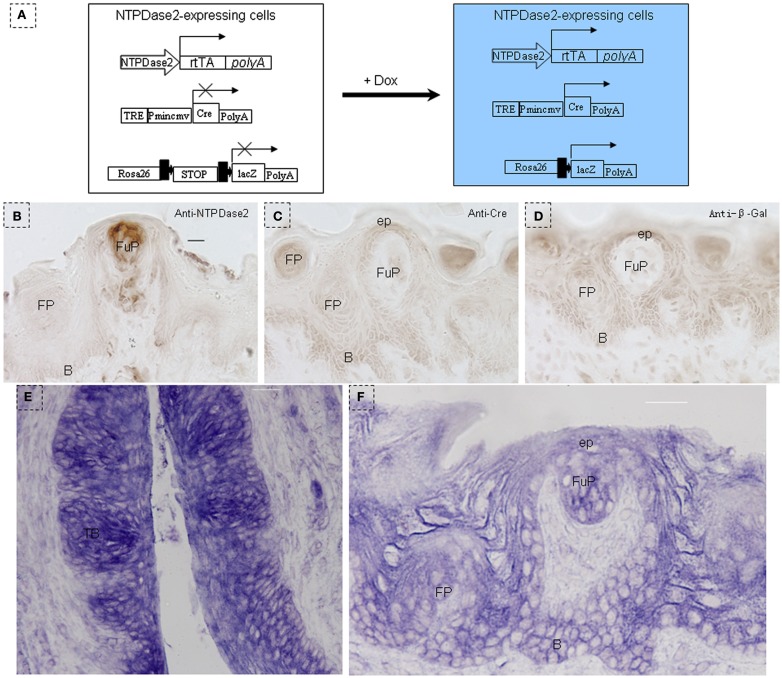
**Identification of NTPDase2-rtTA transgenic mice**. **(A)** Map of NTPDase2-rtTA transgene construct. NTPDase2-rtTA transgenic mice were crossed with TeT-O-Cre and Rosa-LacZ transgenic mice to obtain triple-transgenic mice. After 10 days of Dox treatment of these triple-transgenic mice, expression of the transgene was analyzed by immunohistochemistry. **(B)** Immunohistochemical staining with anti-NTPDase2 revealed that NTPDase2 was expressed in lingual epithelia and papillae. **(C)** Immunohistochemical staining with anti-Cre revealed that Cre was expressed in lingual epithelia and papillae. **(D)** Immunohistochemical staining with anti-β-Gal detected LacZ in lingual epithelia and papillae. *In situ* hybridization with the NTPDase2 probe further revealed the expression of NTPDase2 in lingual epithelia and lingual papillae. **(E)** CV papillae, **(F)** fungiform papillae. B, basal cell layer; SB, suprabasal cell layer; CV, circumvallate papillae; FP, filiform papillae; FuP, fungiform papillae; ep, epithelia; TB, taste bud. Scale bar: **(B–D)**, 12 μm; **(E,F)**, 20 μm.

We generated the NTPDase2-rtTA fusion construct by integrating the *rtTA* gene into the coding sequence of *NTPDase2* (Figures 2A,B). Progeny resulting from the pronuclear (C57/B6) injection of this gene construct were screened for successful integration of the transgene, and transgenic lines were established from one founder mouse. In order to check the expression pattern of the *rtTA* gene, we crossed NTPDase2-rtTA mice with TetO-Cre and RosALacZ mice to obtain the triple-transgenic strain (NTPDase2-rtTA-TetO-Cre-RosALacZ, Figure 3A). Those mice were induced with Doxycycline (Dox) for 10 days (Figure 2C). Immunohistochemical analysis with anti-NTPDase2 showed that NTPDase2 was expressed in the taste buds, as well as lingual epithelia (Figure 3B). Accordingly, we also observed the expression of Cre (Figure 3C) and β-Gal (Figure 3D) in lingual epithelia and taste buds. *In situ* hybridization was also used to further confirm the expression of the *NTPDase2* gene, and as expected it was expressed not only in lingual epithelia but also in lingual papillae (Figures 3E,F). Finally, the NTPDase2-rtTA transgenic mice were crossed with TetO-DTA transgenic mice (Lee et al., 1998) and examined the tongue epithelia in the offspring. After 15 days of Dox induction, NTPDase2+ cells were completely ablated in lingual epithelia and filiform papillae (unpublished data, FL).

**Figure 4:**
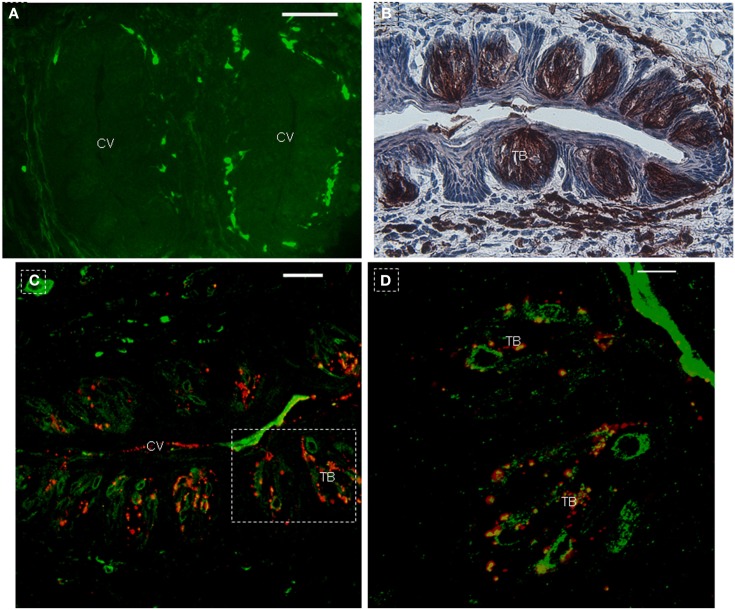
**Co-expression of GFP with GLAST in taste buds after 10 days of Dox induction**. **(A)** Immunohistochemistry with anti-NTPDase2 showed the expression of NTPDase2 in taste buds and connective tissue around CV papillae. **(B)** After 10 days Dox induction, GFP is observed around the CV papillae. **(C)** GFP co-expression with GLAST in taste buds. **(D)** High magnification of taste bud from dotted frame in **(C)**. CV, circumvallate papillae; TB, taste bud. Scale bar: **(A–C)**, 50 μm; **(D)**, 20 μm.

In order to further investigate the expression of the *NTPDase2* gene in lingual epithelia and lingual papillae, we crossed NTPDase2-rtTA mice with a transgenic mouse line carrying the EGFP-pBi-ΔTgfbr2 construct (PTR; Frugier et al., 2005). The double-transgenic NTPDase2-rtTA-PTR mice specifically express the dominant negative ΔTgfbr2 and GFP upon treatment with Dox in the NTPDase2+ cells. After 10 days of Dox induction, we observed obvious expression of GFP in the connective tissue around the CV papillae (Figures 2D and 4A). Furthermore, immunohistochemical analysis with anti-NTPDase2 showed that NTPDase2 was still expressed in the taste buds, as well as connective tissue (Figure 4B). Meanwhile, different pathological changes were observed along the whole tongue (FL, unpublished). In order to determine whether the *rtTA* gene is expressed in taste buds of these mice since NTPDase2 is thought as a marker of type I cells, we carried out immunostaining with anti-GFP. Confocal analysis revealed the expression of GFP in taste buds (Figures 4C,D). The GFP expression, enlarged in Figure 4D, was co-localized with GLAST, which agreed with a previous report of NTPDase2 localizing to the same membrane as GLAST in taste buds (Bartel et al., 2006).

**Figure 5:**
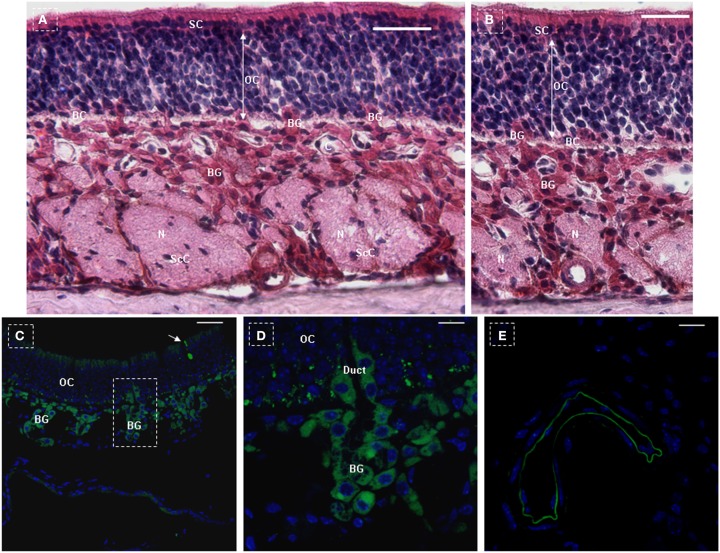
***NTPDase2* and *rtTA* gene expression analysis in main olfactory epithelia of NTPDase2-rtTA-PTR mice**. The three principle cell types of the olfactory epithelium were observed based on the nuclear location and appearance, at low magnification **(A)** and higher magnification **(B)**. The nuclei of the supporting cells (SC) were located closest to the epithelial surface. The SC extended from the basement membrane through the full thickness of the epithelium. The nuclei of the olfactory receptor cells (OC) were immediately beneath the SC. These bipolar neuronal cells localized to different levels within the thickness of the epithelium. The basal cells (BC) were irregularly spaced, characterized by their small round nuclei and were in proximity to the basement membrane. The lamina propria contained olfactory nerves (N) and olfactory (Bowman’s) glands (BG). The duct elements of the Bowman’s glands passed directly through the epithelium to deliver their secretions at the surface. The axons of the olfactory cells were ensheathed by Schwann cells to form the prominent olfactory nerves (N). The nuclei observed within the olfactory nerves represented Schwann cell nuclei (ScC). C, capillaries. Immunohistochemical analysis with anti-NTPDase2 detected NTPDase2 expression in SC, Bowman’s gland, and blood vessels (capillaries). After 10 days of Dox treatment, GFP expression was observed in SC at the surface of the epithelium and sites close to the basal membrane. **(C,D)** Meanwhile, GFP expression was found in both the secretory portion of Bowman’s gland and the duct element extending into the epithelium. **(C,D)** In addition, GFP expression was also observed in the sparse olfactory cells, which extended into the surface of the epithelium [**(C)** arrow]. As expected, GFP expression was detected in blood vessels (capillaries). Scale bar: **(A,B)**, 20 μm; **(C)**, 50 μm; **(D,E)**, 10 μm.

In order to further verify this system (NTPDase2-rtTA-PTR) and determine whether the *rtTA* gene is completely driven by the *NTPDase2* promoter, we analyzed the expression of NTPDase2 and GFP in the main olfactory epithelia after 10 days of Dox induction. In a previous study, it was reported that activity of 5′-nucleotidase is associated specifically with the dark/horizontal basal cells, microvillar cells dispersed at the lumenal side of the epithelium and ducts of Bowman’s gland (Braun and Zimmermann, 1998). Immunohistochemical analysis with anti-NTPDase2 revealed that NTPDase2 was expressed in the lumenal surface of the epithelium, the ducts of Bowman’s gland, Bowman’s gland underneath the basal lamina and Schwann cells around the olfactory nerve bundle (Figures 5A,B). NTPDase2 expression, enlarged in Figure 5B, was also observed at the basal lamina. After 10 days of Dox induction, obvious GFP expression was observed at the lumenal surface of the epithelium, the ducts of Bowman’s gland, Bowman’ gland underneath the basal lamina and the basal cell layer (Figures 5C,D). Meanwhile, we also observed a sparse distribution of GFP+ olfactory cells extending into the epithelial surface (Figure 5C). In addition, GFP was observed at the inner surface of the blood vessels (Figure 5E), similar to the reports that NTPDase2 is expressed in the adventitial cells of blood vessels in other organs (Dranoff, 2002; Sevigny et al., 2002; Vlajkovic et al., 2002; Kishore et al., 2005). Taken together, the above results indicate that rtTA, driven by NTPDase2, may be expressed constitutively and exclusively in the adult tongue.

**Figure 6:**
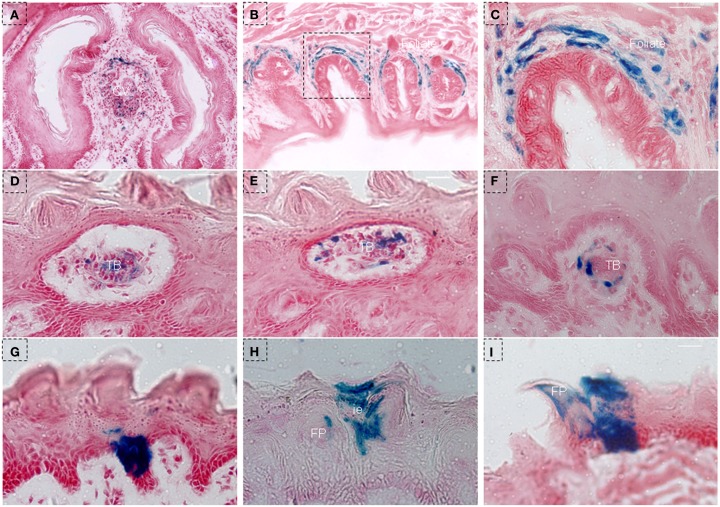
**LacZ staining in lingual epithelia and papillae after 10 days of Dox induction in triple-transgenic mice (NTPDase2-rtTA-TeTO-Cre-RosaLacZ)**. **(A)** LacZ staining (blue) was observed in connective tissue near the CV papillae. **(B)** LacZ staining was detected in connective tissue around foliate papillae. **(C)** High magnification of foliate papillae from the dotted frame in **(B)**. **(D,E)** LacZ staining was observed in taste buds in serial horizontal sections of fungiform papillae. LacZ staining was observed in the developmental papillae **(F)**, the area between the arm of the U-shaped connective tissue core **(G)**, interpapillary epithelium **(H)** filiform papillae and lingual epithelia **(I)**. CV, circumvallate papillae; FP, filiform papillae; FuP, fungiform papillae; ie, interpapillary epithelia; TB, taste bud. Scale bar: **(A,B)**, 125 μm; **(C–I)**, 12 μm.

In order to further investigate the characteristics of NTPDase2+ cells, we treated triple-transgenic mice with Dox using a different schedule (NTPDase2-rtTA-TetO-Cre-RosALacZ, Figures 3A and 2C) and traced the fate of NTPDase2+ cells. After 10 days of Dox induction, blue staining was observed in the connective tissue near the CV papillae (Figure 6A) and the connective tissue around the foliate papillae (Figures 6B,C). The blue LacZ staining was observed in the taste buds of fungiform papillae (Figures 6D,E) and developmental papillae (Figure 6F). More importantly, the blue LacZ staining was found in the area between the arms of the U-shaped connective tissue core (Figure 6G) and interpapillary epithelium (Figure 6H). Meanwhile, LacZ staining was also detected in filiform papillae (Figure 6I).

**Figure 7:**
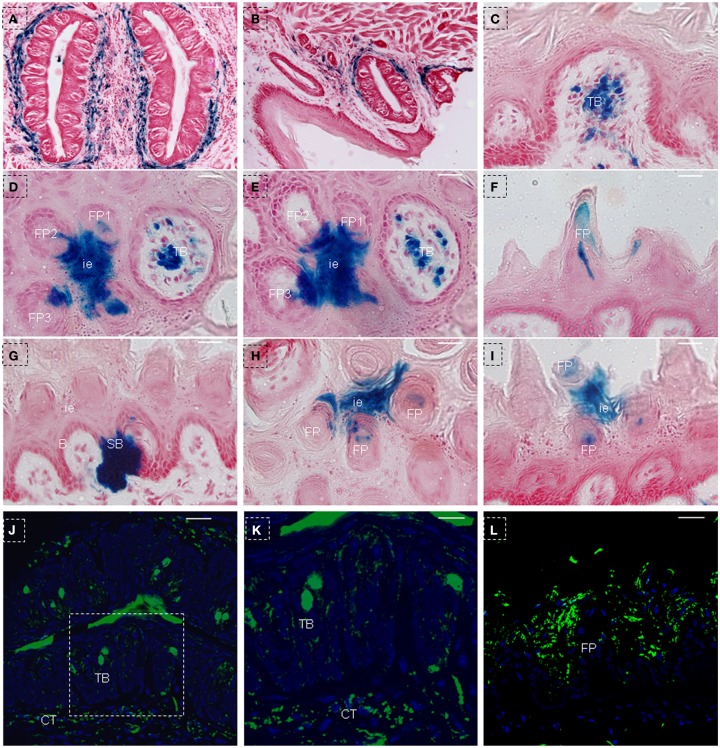
**LacZ staining in lingual epithelia and papillae after 150 days of Dox induction in triple-transgenic mice (NTPDase2-rtTA-TeTO-Cre-RosaLacZ)**. LacZ staining (blue) was detected in connective tissues around CV papillae **(A)** and foliate papillae **(B)**. LacZ staining was observed in the developmental fungiform papillae **(C)**. Serial horizontal sections showed LacZ staining in interpapillary epithelium, filiform papillae and fungiform papillae **(D,E)**. In frontal sections, LacZ staining was detected in the spine of filiform papillae **(F)**, the area between the arm of the U-shaped connective tissue core, and the basal cell layer related to this arm **(G)**. In horizontal sections, LacZ staining was seen in interpapillary epithelium and spine of filiform papillae **(H)**. In frontal sections, LacZ staining was found in the spine of filiform papillae and interpapillary epithelium **(I)**. Immunostaining with anti-β-Gal revealed positive cells in taste buds and connective tissue around the CV papillae **(J)**. **(K)** High magnification of taste bud from the dotted frame in **(J)**. Immunostaining with anti-β-Gal revealed positive cells in the lingual epithelia and filiform papillae **(L)**. B, basal cell layer; SB, suprabasal cell layer; CT, connective tissue; CV, circumvallate papillae; FP, filiform papillae; FuP, fungiform papillae; ie, interpapillary epithelia; TB, taste bud. Scale bar: **(A,B)**, 125 μm: **(C–I)**, 12 μm; **(J,L)**, 50 μm; **(K)**, 20 μm.

In order to further characterize the NTPDase2+ cells, we treated triple-transgenic mice with Dox for a longer period of time (Figure 1C). After 150 days of Dox induction, as expected, the blue LacZ staining was observed around CV papillae (Figure 7A) and foliate papillae (Figure 7B). Notably, we failed to observe the LacZ staining in taste buds of CV and foliate papillae (Figures 7A,B), while it was found in taste buds of fungiform papillae (Figures 7C–E). Meanwhile, LacZ staining was observed not only in the interior and external cells of filiform papillae, but also in the interpapillary epithelia (Figures 7D,E). In frontal sections, the LacZ staining appeared at the spine and middle section of filiform papillae (Figure 7F). In addition, as observed in mice after 10 days of Dox induction, the LacZ staining was seen crossing through the basal cell layer and extending into the suprabasal cell layer, occupying the whole area between the arm of the U-shaped connective tissue core (Figure 7G). Unlike in mice that had received 10 days of Dox induction, we observed many LacZ stained cells distributed at the interpapillary epithelia and the spine of filiform papillae (Figures 7H,I). The above LacZ staining pattern further verified the model of cell migration stream, in which previously proposed that there are multiple populations of stem cells and differentiated cells in lingual epithelia and papillae (Li and Zhou, 2012).

After comparing the blue LacZ staining between the taste buds in CV papillae and fungiform papillae, we speculated that the heavy methylation of the lacZ-coding sequence during cell development/differentiation may have contributed to the absent or lower expression level of LacZ gene, as previously suggested in R26LacZ mice (Chevalier-Mariette et al., 2003; Yamazaki et al., 2005; Foster et al., 2008). Therefore, confocal analysis with anti-β-Gal was used to check the expression of the *lacZ* gene in taste buds of CV papillae and filiform papillae. After examining the sections from mice that had received 150 days of Dox induction, we unexpectedly found that β-Gal positive cells were distributed throughout the whole taste bud (Figures 7J,K), and not limited to just the basal or upper part of the taste bud. As expected, we detected positive signals in the connective tissue around the CV papillae (Figures 7J,K), lingual epithelia (Figure 7L), and filiform papillae (Figure 7L).

**Figure 8:**
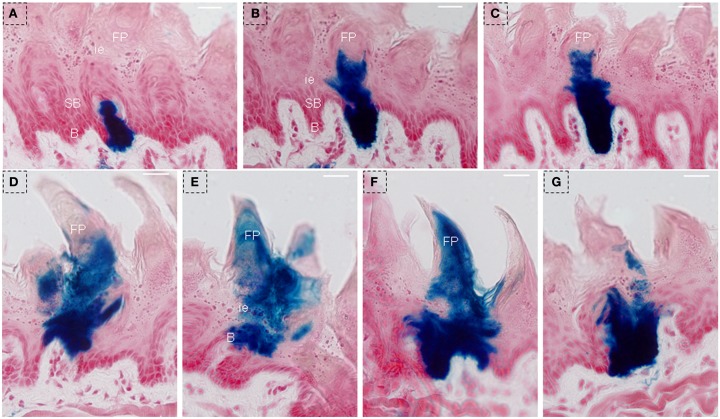
**LacZ staining was observed to extend into the whole lingual epithelia and filiform papillae after 150 days of Dox induction in triple-transgenic mice (NTPDase2-rtTA-TeTO-Cre-RosaLacZ)**. Serial frontal sections showed LacZ staining (blue) occupied the whole lingual epithelia, extending from the basal cell layer into filiform papillae. The diameter of this cluster diameter is ∼60–70 μm **(A–C)**. Serial frontal sections showed LacZ staining across the lingual epithelia, extending into the whole filiform papillae. The diameter of this cluster is ∼80–90 μm **(D–G)**. B, basal cell layer; SB, suprabasal cell layer; CT, connective tissue; FP, filiform papillae; FuP, fungiform papillae; ie, interpapillary epithelia; TB, taste bud. Scale bar: 12 μm.

Two clusters of blue staining in lingual epithelia were shown by serial frontal section from triple-transgenic mice received 150 days of Dox induction. One’ diameter was ∼60–70 μm, and blue staining occupied the whole lingual epithelia, extending from the basal cell layer into filiform papillae (Figures 8A–C). Another’ diameter was ∼80–90 μm, and blue staining was across the lingual epithelia, extending into the whole filiform papillae (Figures 8D–G).

**Figure 9:**
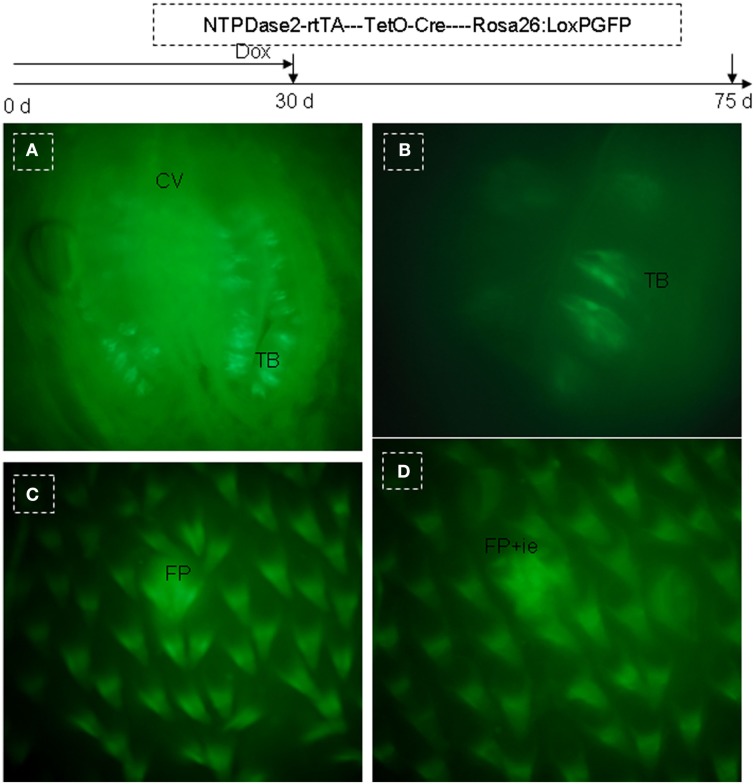
**Genetic tracing of NTPDase2+ cells in the triple-transgenic mouse system (NTPDase2-rtTA-TeTO-Cre-RosaGFP)**. A triple-transgenic mouse was treated with Dox for 30 days and then sacrificed 45 days later to examine GFP expression. **(A)** GFP was observed in CV papillae. **(B)** High resolution image of a taste bud. **(C)** GFP was observed in filiform papillae. **(D)** GFP was observed in filiform papillae and interpapillary epithelium. CV, circumvallate papillae; FP, filiform papillae; ie, interpapillary epithelia; TB, taste bud.

In order to further test the above hypothesis, we generated another triple-transgenic mouse strain, NTPDase2-rtTA-TetO-Cre-RosAGFP, with GFP as a marker. After 15 or 30 days of Dox induction, the Dox treatment was stopped, and mice were sacrificed to observe GFP expression after another 40–45 days. Previous studies have suggested that the turn-over time of taste bud cells is ∼10 days (Beidler and Smallman, 1965; Kinnamon et al., 1985; Delay et al., 1986) and ∼1–3 days in lingual epithelia (Cameron, 1966) in adult mice. Thus, the period of 40–45 days is sufficient to clear out the originally labeled cells both in lingual papillae and lingual epithelia. After directly observing the tongue, we found obvious GFP expression on the dorsal surface in mice after receiving either 15 days (data not shown) or 30 of days Dox induction. As expected, we clearly observed GFP expression in taste buds of CV papillae (Figures 9A,B). Meanwhile, GFP expression was also found in filiform papillae (Figure 9C) and interpapillary epithelia (Figure 9D).

## Discussion

The morphological development of fungiform papillae is characterized first by an epithelial thickening, with subsequent epithelial-mesenchymal interactions to form the connective tissue core surrounded by an annular downgrowth of epithelium (Farbman, 1965a,b). Fungiform papillae cannot regenerate in the absence of connective tissue cores (Hellekant et al., 1987). Although non-gustatory oral epithelia or epidermis can be transplanted into regions innervated by taste nerve fibers, taste buds cannot be induced in these epithelia even in the presence of nerves that have the capacity to induce them in appropriate epithelia (Zalewski, 1972). However, Wright (1951, 1958, 1964) used roles of different ages to show that tongue transplanted to the orbit, side of the body, tail, or surface of the liver would first lose taste buds, but then the buds would reappear after several days. These observations collectively indicate that certain factors may be pre-stored in lingual epithelia and lingual papillae to facilitate this regeneration, as suggested that placodes go through morphogenesis to form the taste papillae with interaction to a mesenchymal core from embryonic day 12 to post natal day 4 in mice (Thirumangalathu and Barlow, 2009; Thirumangalathu et al., 2009), and since then, a population of taste bud stem cells is established within the local epithelium via local cell–cell interactions (Barlow et al., 1996; Barlow and Northcutt, 1997). We have previously proposed a cell migration stream existing at the lingual epithelia and filiform papillae (Li and Zhou, 2012), and K14+ lineage cells are known to contribute to the development of lingual epithelia and papillae (Okubo et al., 2009; Li and Zhou, 2012). Here, the current results demonstrated the generation of lingual epithelia and papillae from NTPDase2+ cells. In fact, our results were not surprising, for it has been suggested that common progenitor cells may contribute to taste cells and epithelial cells, and it has been further pointed out that taste receptor cells originate from the local epithelium as well (Stone et al., 1995). On the other hand, type I cells express GLAST and NTPDase2, suggesting they have glial-like functions, such as transmitter clearance and functional isolation of other taste cell types (Finger, 2005). Interestingly, we also observed NTPDase2 expression in supporting cells and Bowman’s glands of olfactory epithelia, indicating that NTPDase2+ cells may carry out glial functions, similar to olfactory receptor and sustentacular supporting cells that are activated by ATP in the olfactory epithelium of *Xenopus laevis* tadpoles (Czesnik et al., 2006). On the other hand, it should be noted that the nature of NTPDase2+ cells revealed in the current study is similar to the context in which these cells are found in neural stem cell (NSC) of the SVZ (Braun et al., 2003; Mishra et al., 2006; Zimmermann, 2006). NTPDase2+ cells are selectively linked to the type-B cells (adult NSC), absent in type-C cells (TACs), and type-A cells (migrating neuroblasts). Intriguingly, NTPDase2+ cells are associated with astrocyte-like cells ensheathing the migrating neuroblasts within the rostral migratory stream (RMS).

**Figure 10:**
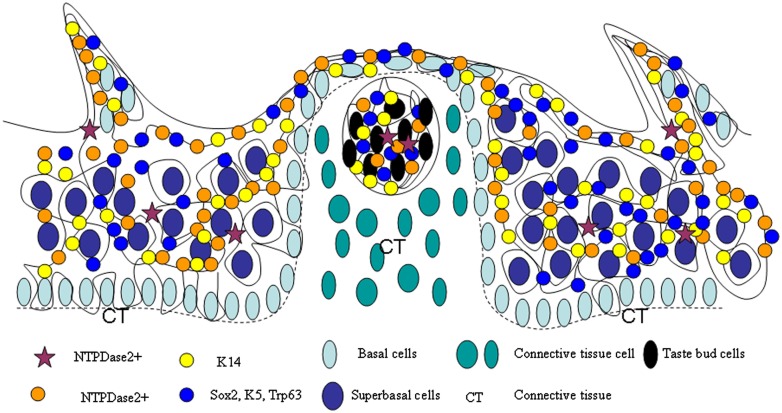
**Model of lingual epithelia cell genesis**. Representation of a cell migration stream in lingual epithelia and lingual papillae, which is comprised of multiple stem cell populations, such as those expressing NTPDase2, K14, Sox2, K5, and Trp63.

Several findings presented here support the hypothesis (Okubo et al., 2009; Li and Zhou, 2012) that there is a cell migration stream in tongue epithelia consisting of stem cell pools, TA cell pools and differentiation cell pools (Figure 10). The many cell pools of the cell migration stream include among others the NTPDase2, K14, Sox2, K5, and Trp63 expressing populations. Local micro-environments provide an important cue for cell development/differentiation. Different epidermal stem cell populations have also been reported in adult mouse hair follicles and interfollicular epidermis (Jensen et al., 2009; Watt and Jensen, 2009). On the other hand, the cell migration stream has been found in several types of stratified epithelia. In the interfollicular epidermis, the cell migration stream has previously been shown from the bulge region to the bulb of the hair follicle (Oshima et al., 2001). Intestine stem cells reside in the base of the crypt, These stem cells are thought to move upward to generate proliferative transiently amplifying (TA) cells, which continually differentiate upward into three lineages, enteroendocrine cells, goblet cells, and enterocytes, to form the villus (Barker et al., 2007; Blanpain et al., 2007; Sato et al., 2011; Ordonez-Moran and Huelsken, 2012). The corneal epithelium is another stratified epithelium. The limbus region is thought to hold corneal stem cells, which gives rise to TA cells that migrate toward the center of the cornea (Sun and Lavker, 2004). Thus, it is conceivable that the existence of the cell migration stream may be a common feature for diverse stratified epithelia.

## Materials and Methods

### Generation of NTPDase2-rtTA transgenic mice and Dox treatment

The expression construct was generated by cloning the *rtTA* gene and its polyadenylation signal into RP23-47P18 (http://bacpac.chori.org), replacing the coding sequence of *NTPDase2*, using Red recombination system. In order to verify the exact insert by pulse-field gel electrophoresis (PFGE), *Sal*I and *Xho*I was introduced downstream of the *rtTA* gene. The inserted fragment was finally verified by sequencing. Transgenic lines were produced by pronuclear injection of zygotes from C57BL/6 mice using the modified RP23-47P18. TetO-EGFP-Tgfbr2 (PTR), TetO-Cre, TetO-DTA, Rosa26-flox-STOP-flox-LacZ, Rosa26-flox-STOP-flox-GFP animals were obtained from Jackson Laboratories (Bar Harbor, ME, USA). Those transgenic mice were bred and maintained at the Monell Chemical Senses Center animal facility. All procedures involving animals were approved by the Monell Chemical Senses Center Institutional Animal Care and Use Committee.

Doxycycline administration: Doxycycline (Sigma, St. Louis, MO, USA) was diluted in 5% sucrose in water to a final concentration of 0.3–0.5 mg/ml and supplied as drinking water. Access to the Dox-containing water was unlimited and was changed every 2 ± 3 days. A single intraperitoneal injection of Dox (10 mg/kg body weight) was also administered while the mice began receiving Dox-containing water.

### Histology and immunostaining

For immunocytochemistry, mice were perfused transcardially with 2–4% paraformaldehyde (PFA) in phosphate-buffered saline (PBS; pH 7.2–7.4). The tongue tissues were dissected, post-fixed in PFA for 2–12 h and cryoprotected in 30% sucrose in PBS at 4°C overnight. After sectioning on a cryostat, 10–12 μm sections were collected onto Superfrost Plus Microscope slides (Fisher Scientific). Polyclonal primary antibodies were against NTPDase2 (Sevigny, 2002), β-Galactosidase (chicken, Abcam, ab-9361), Cre (rabbit, Novagen, 69050), GFP (goat, Abcam, ab-5450; rabbit Abcam, ab-6556), Glast (Pig, Chemicon, AB1782), Sox2 (rabbit, Abcam, ab-59776), PLCβ2 (rabbit, Santa Cruz Biotechnology, sc-206), and GNAT3 (rabbit, Santa Cruz Biotechnology, sc-395). Staining was performed with the TSA Plus system from PerkinElmer according to the manufacturer’s instructions. Fluorescent images were captured with the Leica TCS SP2 Spectral Confocal Microscope (Leica Microsystems Inc., Mannheim, Germany).

Stainings against NTPDase2, Cre, and β-Galactosidase were performed with standard immunocytochemical procedures according to the manufacturer’s instructions (VECTASTAIN Elite ABC Kits).

### LacZ staining

Animals were perfused with 2% PFA in PBS). The tongues were removed and fixed in 2% PFA for 1 h, after which it was cryoprotected in 30% sucrose in PBS at 4°C overnight. The following morning, the tongue tissue was cryosectioned at 20 μm thickness. The sections were then washed three times (3 × 20 min) in PBS, followed by staining in X-gal solution (5 mM potassium ferrocyanide, 5 mM potassium ferricyanide, 2 mM MgCl_2_, 0.02% NP-40, 0.01% Na deoxycholate, 1 mg/ml X-gal) at 37°C overnight. The stained sections were washed three times (3 × 20 min) in PBS and counterstained with nuclear fast red. Brightfield images were captured using a SPOT digital camera (Diagnostic Instruments, Inc.) attached to a Nikon SA Microphot microscope and minimally processed using Image-Pro Plus image analysis software.

### *In situ* hybridization

*In situ* hybridization was performed as previously described (Ishii et al., 2004). Sections were hybridized with digoxigenin-labeled RNA probes. Probes were prepared from PCR products that were subcloned into pBluescript (+) and labeled with digoxigenin-UTP by *in vitro* transcription with T7 RNA polymerase. The following primers were used for PCR amplification of a 487 bp fragment of *NTPDase2*: forward primer, 5′-TGCCTGTGGGAACCCTGAA-3′; reverse primer 5′-GACCGTCCAGGCTGCTTCA-3′.

## Conflict of Interest Statement

The authors declare that the research was conducted in the absence of any commercial or financial relationships that could be construed as a potential conflict of interest.

## Abbreviations

CV, circumvallate; K14, Keratin 14; NSC, neural stem cell; NTPDase2, ecto-nucleoside triphosphate diphosphohydrolase 2; PTR, TetO-EGFP-ΔTgfbr2; SVZ, subventricular zone; TACs, transit amplifying cells; TB, taste buds; Tgfbr2, TGF-β receptor type II; TGF-β, transforming growth factor β.
